# Anaesthesia Management for Neurosurgery in a Patient With Congenital Lung Agenesis

**DOI:** 10.7759/cureus.54522

**Published:** 2024-02-20

**Authors:** Sangeetha Selvaraj, Ying Mao Gn, Theodore G Wong, Shariq Ali Khan

**Affiliations:** 1 Anaesthesiology, Singapore General Hospital, Singapore, SGP

**Keywords:** computed tomography, barotrauma, lung, bronchus, trachea, neurosurgical procedures, lung agenesis

## Abstract

Congenital lung agenesis is a rare congenital abnormality associated with an absence or under-development of either one or both lungs, and its presentation in adulthood is even rarer.

We describe a 40-year-old female patient with a history of congenital agenesis of the right lung and a high-grade glioma in the frontal region of the brain presenting for craniotomy and excision of the tumor in an MRI suite. Lung protective strategies of ventilation were utilized intraoperatively. The remote location of the MRI suite made access to extra manpower support challenging. The patient was managed uneventfully and discharged stable to the high-dependency unit.

Our case describes how congenital lung agenesis poses a unique set of challenges for anaesthetic management, particularly in neurosurgical patients, and provides guidance to a multidisciplinary team approach.

## Introduction

Pulmonary agenesis represents a complete lack of bronchi, pulmonary vasculature, and lung tissue. It is a rare congenital defect, with an incidence of approximately 1 in 100,000 births [1]. It can be either unilateral or bilateral, the bilateral type being incompatible with extra-uterine existence. The prognosis is not favourable for unilateral right lung agenesis because of the often present cardiovascular abnormalities and the increased degree of mediastinal shift that causes compression and distortion of the trachea and mediastinal vessels [2,3]. Over 50% of the cases die in the first five years of life [4,5]. On the other hand, the illness seldom manifests as an adult or stays asymptomatic throughout life [6]. We present to you one such rare case of a woman with right lung agenesis presenting in her adulthood. Stable hemodynamics and ventilatory management were crucial in this case due to neurological pathology that required neurosurgery, in addition to the potential systemic problems attributed to lung agenesis.

## Case presentation

**Figure 1:**
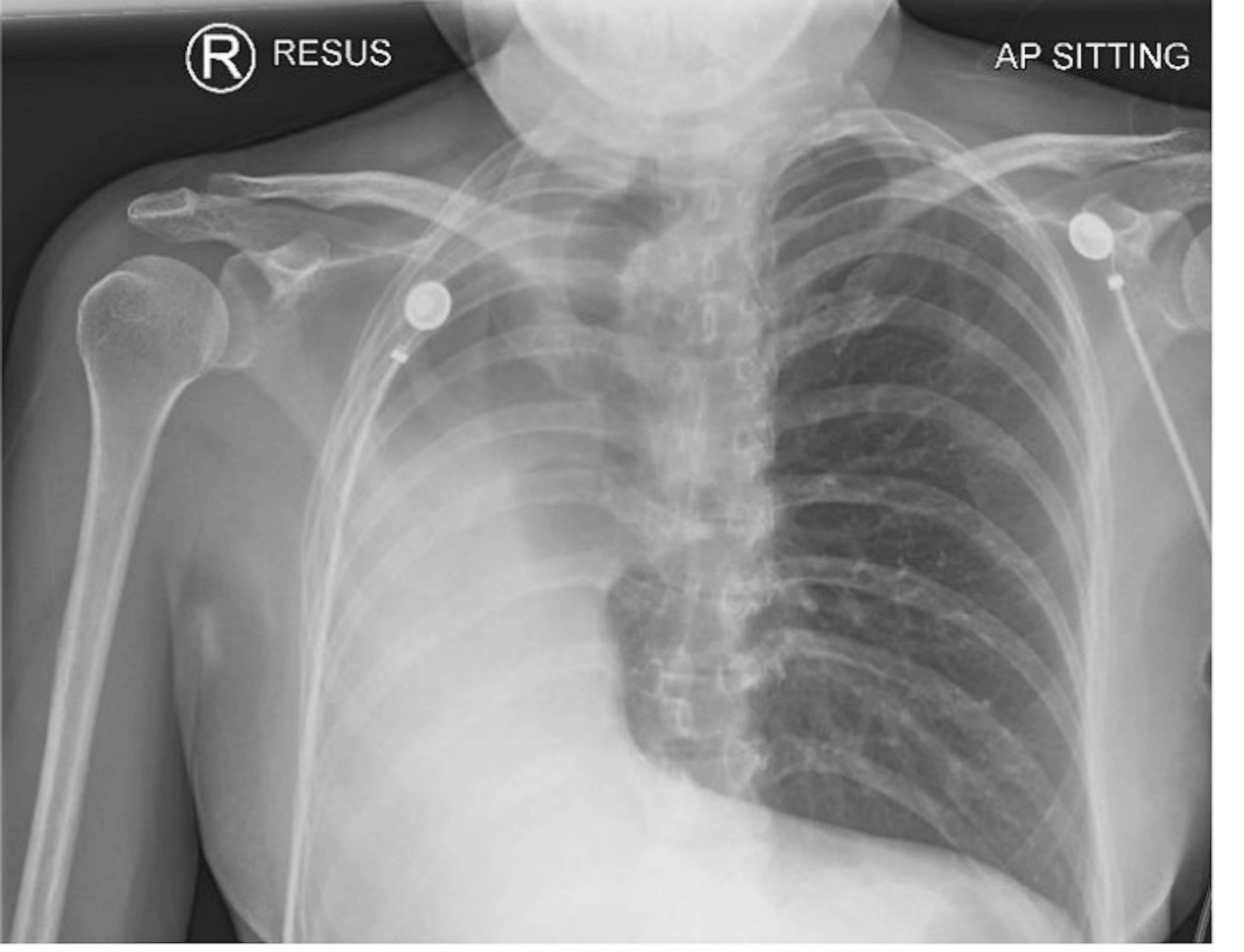
CXR shows the absence of the right lung with severe ipsilateral mediastinal shift with compensatory hyperinflation of the left lung with herniation into the right hemithorax. There is marked tracheal deviation due to right mediastinal shift.

**Figure 2:**
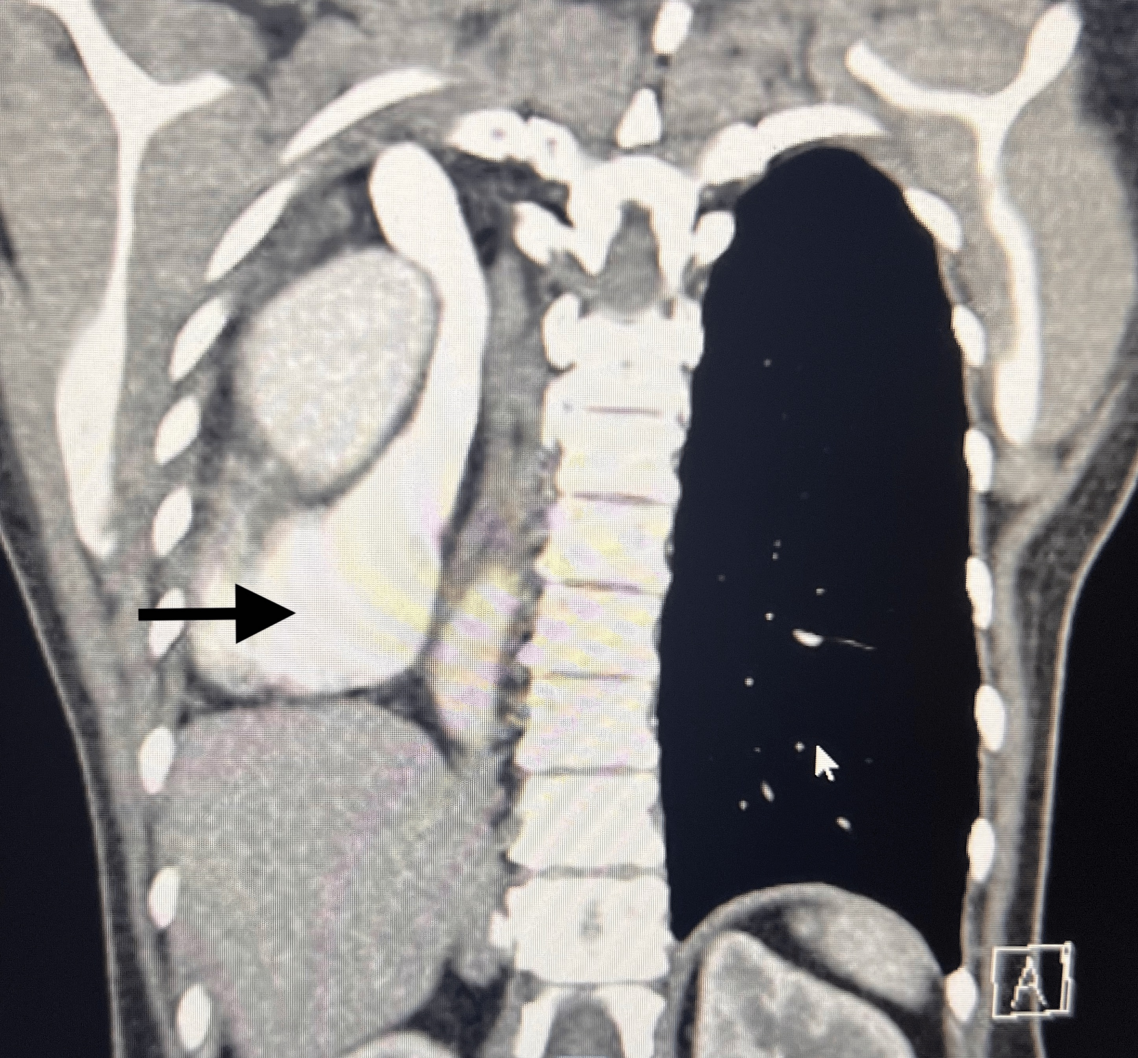
CT chest showing mediastinal structures and the heart lying in the right hemithorax.

**Figure 3:**
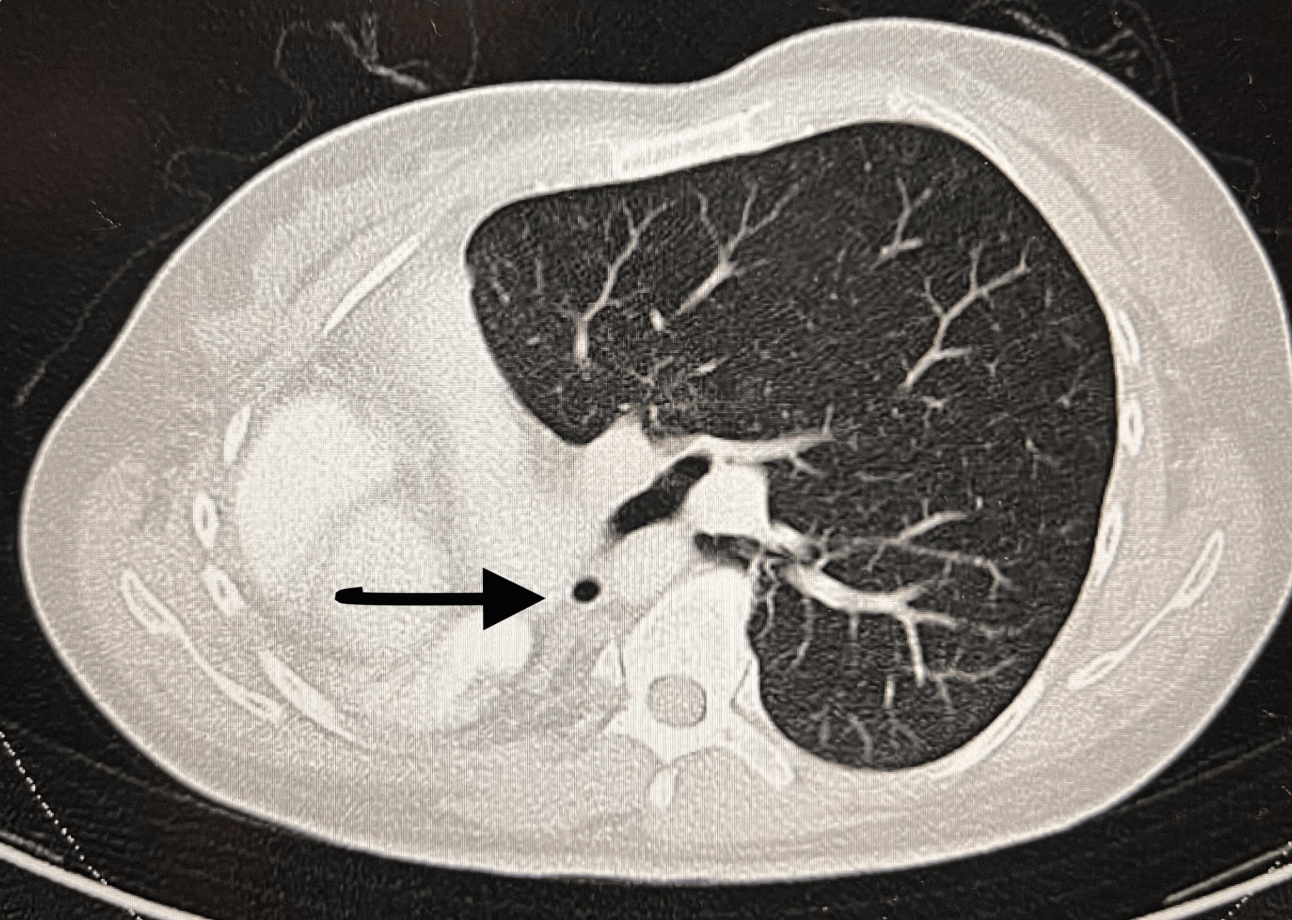
CT chest showing the stump of the right bronchus and absence of the right lung.

**Figure 4:**
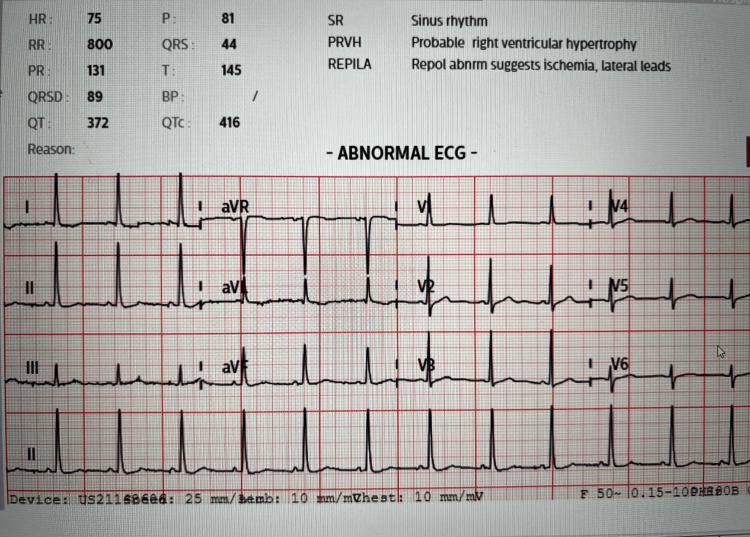
ECG showing normal sinus rhythm with the shift of transitional zone of the QRS complex in the chest leads to the right, compatible with the right mediastinal shift.

Written informed consent was obtained from the patient. A 40-year-old female presented with a three-month history of non-vertiginous giddiness associated with episodic blurring of vision and nausea with vomiting. She also reported a history of right lung agenesis which was diagnosed at three years of age arising from lung imaging performed due to a history of recurrent chest infection. The frequency of her chest infections improved after 12 years of age and was not on any follow-up since 2017. She did not give any history of recurrent hospital admissions and was not on any chronic medications. She described good effort tolerance and went hiking on a weekly basis. She denied any history of giddiness, syncope, palpitations, orthopnoea, or paroxysmal nocturnal dyspnoea which might be suggestive of cardiac failure. In view of her neurological symptoms, an MRI brain was performed which showed a large heterogenous intra-axial mass in the right frontal lobe suspicious of high-grade glioma and was planned for a craniotomy and excision of the right frontal lesion in the MRI suite. On examination, the patient did not have any dysmorphic features, was comfortable, not in respiratory distress, and speaking in full sentences. She was not tachypneic, and her respiratory rate was 12 breaths/min. Examination of the chest revealed decreased chest excursions, absent air entry, and absent breath sounds along the right hemithorax. Cardiac sounds were heard along the right hemithorax. SpO2 was 97% on room air. Her upper airway examination was unremarkable. Figures 1 is the CXR image, and Figures 2-3 are the CT images that have been elaborated subsequently. Figure 4 is the ECG image showing changes secondary to the right mediastinal shift.

MRI of the cervical spine showed left convexity cervical spine scoliosis. Her blood results showed normal electrolytes (sodium 139 mmol/l, potassium 4.3 mmol/l, bicarbonate 24.2 mmol/l). Since her condition remained stable, a blood gas analysis wasn't performed. No pre-existing lung function test was performed. She was referred to a respiratory medicine physician and a cardiologist for preoperative optimization. Transthoracic echocardiography was performed, which did not report any structural abnormality; ejection fraction was preserved, and pulmonary artery systolic pressure was within normal limits. She was deemed fit to proceed from a respiratory point of view, as she was functionally well-compensated. No elevated cardiac risks were quoted secondary to the dextrocardia. Her history was substantially discussed with the neurosurgeon due to concerns about difficulty in obtaining help in the MRI suite in the event of intraoperative anaesthetic complications. However, it was deemed necessary by the surgeon for the operation to be performed in the MRI suite to determine the adequacy of resection margins intraoperatively. A team brief was performed at the start of the case, and a contingency plan was communicated to all members of the medical team to minimize delay in response should a crisis arise intraoperatively. On the day of surgery, standard ASA (American Society of Anesthesiologists) monitors and an intra-arterial line were placed prior to the induction of anaesthesia. She was induced with total intravenous anaesthesia using target-controlled infusion (TCI) propofol and TCI remifentanil infusions. Mask ventilation was attempted before muscle paralysis, and she was intubated uneventfully using a McGrath hyper angulated blade (Medtronic, Dublin, IE) with minimal neck manipulation due to concerns of cervical spondylosis. A left-sided femoral central venous catheter was inserted post-induction in the event of a need for hemodynamic support intraoperatively. Changes in structural characteristics would call for a modification of the airway device. A reinforced endotracheal tube (ETT) would have been perfect in our instance because the mediastinal displacement caused a large tracheal deviation. Kinks are even more likely after lengthy surgeries because the PVC ETT softens. Although reinforced tubes are MRI conditional, we chose to use PVC ETT instead to avoid the rare possibility of tube dislodgement, which worked fine in the end. To minimize the risk of barotrauma, she was ventilated with pressure-controlled ventilation. A tidal volume of 6 ml/kg predicted body weight was achieved with pressure support of 10-15 cm H_2_0, and the peak inspiratory pressure did not exceed 30 cm H_2_0. Her other ventilator settings include a fiO2 (fraction of inspired oxygen) of 0.4-0.5, a positive end-expiratory pressure of 5 cm H_2_0, a respiratory rate of 10/min and a 1:1 inspiration to expiration ratio. Throughout the procedure, she remained hemodynamically stable. A blood gas analysis performed during surgery revealed normal values (PaO2 278 on fiO2 0.4, PaCO2 41.3, SO2 100%, HCO3 23.6, pH 7.364, BE -2). The operation was uneventful, and she was carefully extubated fully awake, and transferred to the neurosurgical intensive care unit for postoperative monitoring. She did not require any supplemental oxygen post-operatively. She had received intensive chest physiotherapy since postoperative day 1 and was subsequently discharged from the hospital on postoperative day 4 uneventfully. On the day of discharge, her vitals remained stable, and she remained afebrile with no complaints of dyspnoea or any other adverse respiratory signs or symptoms.

## Discussion

Asymptomatic patients with congenital lung agenesis who survive to adulthood may be well compensated, but tailored anaesthetic management is imperative to minimize the risk of perioperative complications, which may increase the risk of morbidity and mortality.

Lung agenesis is commonly associated with congenital defects, including cardiovascular, skeletal, gastrointestinal and genitourinary systems. In nearly half of the cases, unilateral lung agenesis is associated with additional abnormalities of the musculoskeletal, gastrointestinal, cardiovascular, or genitourinary systems. Tetralogy of Fallot, aortic coarctation, patent ductus arteriosus, atrial, and ventricular septal defects, tricuspid insufficiency, and abnormal pulmonary venous return are the most often occurring concomitant cardiac defects [5].

A ventral bud, also known as a respiratory diverticulum, emerges from the foregut towards the end of the first month of intrauterine life and elongates and divides to form two lungs. The failure of the division of this primitive bud into two results in the failure of the development of one lung, while the other lung develops normally [7]. Depending on the stage at which the growth process is arrested, there can be complete or partial absence of lung tissue. It has been subdivided into three types according to the development of their primitive lung bud, initially by Schneider and later refined by Boyden: (1) Pulmonary agenesis (type 1): There is total absence of bronchus, lung tissue, and pulmonary vasculature. (2) Pulmonary aplasia (type 2): There is a rudimentary, short, blind-ending main bronchus with absent lung tissue and pulmonary vessels. (3) Pulmonary hypoplasia (type 3): The bronchial tree, pulmonary vasculature, and lung tissue are present in varying degrees. These children typically present with recurring chest infections or respiratory distress. Their heightened susceptibility to lung infections may stem from the modified tracheal mechanics brought about by the mediastinal shift, which causes the trachea to stretch and compress. It is quite rare for presentations to be late.

The presence of these abnormalities may pose additional anaesthetic considerations in perioperative management. These patients may have complications directly arising from lung agenesis, such as restrictive lung disease and pulmonary hypertension [8]. There can be enlargement of the solitary normally developed lung. This expansion of the lung represents true hypertrophy to accommodate the body's physiological needs. There can also be emphysematous changes in normally developed lungs [8], that may increase the likelihood of cardiovascular complications arising from the distortion of the mediastinal structures as they shift to the contralateral side. To prevent life-threatening consequences of mediastinal shift, tissue expanders have been inserted into the empty space [9]. It is essential to demonstrate and report any tracheal or bronchial narrowing or kinking due to the mediastinal shift [10,11].

Thorough physical examination and appropriate radiological investigations are paramount to ascertain the severity of lung pathology. CT of the chest allows visualization of the bronchial tree, parenchyma, and vasculature; hence it is considered to be the ideal diagnostic tool for pulmonary agenesis [4]. Differential diagnoses of opacification of hemithorax would include total lung collapse, diaphragmatic hernia, cystic adenomatoid lung malformations, pulmonary sequestration, scimitar syndrome, and prior pneumonectomy.

Intraoperative management is similar to the management of a post-pneumonectomy patient, including the use of reduced tidal volumes to reduce the risk of barotrauma. Since tissue in pulmonary agenesis is undeveloped and brittle from aberrant blood flow during early embryonic development, it essentially functions as a single lung with friable tissue. Positive pressure ventilation may result in rupture of the bronchial stump, thus increasing the risk of interstitial emphysema, pneumothorax, barotrauma, and pneumomediastinum on the unaffected side [12]. Consequently, a smaller tidal volume and higher respiratory rate lung-protective approach is advised. In cases where the bronchial stump is deemed to have a thin wall, one-lung ventilation has been utilized successfully to minimize the risk of barotrauma to the hypoplastic lung or stump [12,13]. Furthermore, the single lung increases airway resistance, which implies that higher peak pressures are required to overcome the resistance and avoid shunting and hypoxemia. Using a smaller endotracheal tube for airway management due to a stenotic or undeveloped trachea further increases airway resistance. Hence, it is crucial to monitor the airway pressures closely while ensuring adequate minute ventilation. Overdistension of the lung can also worsen the underlying mediastinal shift, resulting in further compression of the mediastinal vessels and hence resulting in hemodynamic compromise. Consideration should also be given to avoid procedures on the ipsilateral side of the sole functioning lung (i.e., insertion of the central venous catheter, performing brachial plexus block) to preserve lung function. Postoperatively, patients with lung agenesis should receive intensive chest physiotherapy, as they are at increased risk of atelectasis and pneumonia.

These ventilatory methods heighten the possibility of a mismatch between ventilation and perfusion, which could contribute to hypoxia and hypercarbia, which might increase intracranial pressure. Hypotension from mediastinal shift or tension pneumothorax must be particularly avoided during neurosurgery, as this increases the risk of inadequate cerebral perfusion. Reduced compliance of the single lung and possible barotrauma can raise intrathoracic pressure, which may consequently lower cerebral venous outflow and increase intracranial pressure. Furthermore, neurosurgical procedures are occasionally performed in the MRI suite, which presents additional challenges due to the potential delay in obtaining help and the incompatibility of the resuscitation equipment within the MRI suite. Therefore, it is crucial to prevent adverse neurological outcomes through careful patient selection, appropriate ventilatory strategies, vigilant monitoring, the use of invasive monitoring for frequent arterial blood gas analysis, and being prepared for potential complications.

## Conclusions

This case report describes the successful anaesthetic management of a patient with congenital lung agenesis coming for neurosurgery. A comprehensive preoperative evaluation and planning, meticulous intraoperative management and postoperative pulmonary care are key to a favourable clinical outcome for the patient. Anaesthesiologists should be aware of the unique challenges posed by this condition, and provide individualized strategies to ensure safe and effective perioperative care.
